# Application of rivaroxaban in patients with non-valvular atrial fibrillation and end-stage kidney disease: A systematic review and meta-analysis

**DOI:** 10.3389/fcvm.2023.1021959

**Published:** 2023-02-07

**Authors:** Zhenzhen Yang, Jieya Wang, Ye Yuan, Tian Cheng, Feifei Ren, Songsong Wang, Zhiqing Zhang

**Affiliations:** ^1^Department of Pharmacy, The Second Hospital of Hebei Medical University, Shijiazhuang, China; ^2^Department of Pharmacy, The Sixth Affiliated Hospital of Kunming Medical University, Yuxi, China; ^3^Department of Pharmacology, Hebei Medical University, Shijiazhuang, China

**Keywords:** rivaroxaban, non-valvular atrial fibrillation, end-stage kidney disease, systematic review, meta-analysis

## Abstract

**Background:**

Nowadays, the number of patients with non-valvular atrial fibrillation (NVAF) complicated by end-stage renal disease (ESKD) is increasing. There are significant challenges in anticoagulation with prescription drugs because of the high risk of bleeding and embolism among these patients. However, no randomized controlled trials (RCTs) of warfarin in combination with any non-vitamin K oral anticoagulant (NOACs) have been performed in patients with baseline creatinine clearance (CrCl) <25 ml/min, which makes it difficult to justify the use of anticoagulants in such patients. Then, we aimed to collect and summarize all evidence to enable the anticoagulation of rivaroxaban, which is less cleared by the kidneys, in patients with severe renal insufficiency and to complement and improve the evidence on the use of rivaroxaban for anticoagulation.

**Methods:**

The present systematic review and meta-analysis searched the databases of *PubMed*, *Embase*, the *Cochrane Library*, *CNKI*, *CBM*, and *Google Scholar* for relevant studies from inception to 1 June 2022, with the restriction of English and Chinese. Eligible cohort studies and RCTs that reported efficacy outcomes [composite of stroke and systemic embolism (SSE), ischemic stroke (ICS), and systemic embolization] or safety outcomes [major bleeding, intracranial hemorrhage (ICH), and gastrointestinal bleeding (GIB)] of rivaroxaban in NVAF patients with ESKD were enrolled. Two authors completed the data extraction and quality assessment work, respectively. The Cochrane Collaboration tool for assessing the risk of bias was used for RCTs, and the NEW-Castle Ottawa scale was used for study quality assessment for cohort studies. Dichotomous variables were calculated as risk factors with 95% confidence intervals (CIs), and meta-analysis was performed to probe the effect of research design, rivaroxaban dose, and controlled drug factors on outcomes.

**Results:**

In total, three studies were included for meta-analysis, involving 6,071 NVAF patients with ESKD, and two studies were included for qualitative analysis. All included studies were at low risk of bias. A meta-analysis demonstrated that mix-dose rivaroxaban caused no statistical discrepancy in the occurrence of thrombotic and bleeding events when compared to the control group (embolism, LogOR: −0.64, 95% CI: −1.05 to −0.23, P:0.25; bleeding, LogOR: −0.33, 95% CI: −0.63 to −0.03, P:0.15), and low-dose rivaroxaban produced similar results (embolism, LogOR: −1.04, 95% CI: −2.15 to 0.07, P:0.61; bleeding, LogOR: −0.81, 95% CI: −1.19 to −0.44, P:0.93).

**Conclusion:**

In this study, low-dose rivaroxaban (10 mg, once a day) may benefit more than warfarin in patients with NVAF and ESKD.

**Systematic review registration:**

https://www.crd.york.ac.uk/prospero/#recordDetails, identifier CRD42022330973.

## Introduction

Atrial fibrillation (AF) is a supraventricular arrhythmia caused by uncoordinated atrial electrical activity followed by ineffective atrial contraction, and it is the most common sustained arrhythmia in adults around the world. Stroke due to AF is usually more symptomatic, higher recurrence rate, and is often fatal or disabling compared to non-cardiac stroke (1). AF and renal insufficiency promote each other and are risk factors for each other. The results of the Atherosclerosis Risk in Communities (ARIC) study suggest that renal function decline is closely related to the incidence of AF and is independent of other risk factors (2). The Chronic Renal Insufficiency Cohort (CRIC) study showed that the prevalence of AF in patients with estimated glomerular filtration rate (eGFR) ≥45 ml/min was 16%; however, the prevalence increased to 20.4% when eGFR <45 ml/min (3). Approximately 13–27% of patients with an estimated creatinine clearance (CrCl) ≤ 15 ml/min have AF, which is significantly higher than that in the general population, and 12% of patients with AF have a CrCl ≤ 15 ml/min or receive dialysis, which means combined with ESKD (4–6).

Non-valvular atrial fibrillation (NVAF) is the exception for AF after severe mitral stenosis or mechanical valve replacement. At present, non-vitamin K antagonist oral anticoagulant (NOAC) has become the first choice of anticoagulant therapy for NVAF. However, in the anticoagulation therapy of patients with NVAF and ESKD, the benefit of NOACs is limited and controversial. In clinical practice, doctors are more inclined to give warfarin to NVAF patients with ESKD because the anticoagulant intensity of warfarin can be judged by monitoring the international normalized ratio (INR). However, a meta-analysis indicated that anticoagulation with warfarin in patients with NVAF and dialysis did not reduce the risk of stroke compared with patients without anticoagulation (7, 8). Other studies have shown that warfarin may even increase the risk of ischemic stroke (ICS) (9, 10). Based on this, it is necessary to further consider the efficacy and safety of NOAC anticoagulation in NVAF patients with ESKD, and it can also provide more evidence for the clinical application of anticoagulants.

Rivaroxaban (10 mg/day) is approved for NVAF patients with CrCl between 15 and 50 ml/min in the Taiwan Province of China and Japan, which may provide evidence for the efficacy and safety of rivaroxaban in patients with ESKD. In this study, we systematically searched the published literature about the application of rivaroxaban for stroke prevention in patients with NVAF and ESKD and performed a quantitative meta-analysis of the retrieved literature to provide a reference for the application of rivaroxaban in the prevention of stroke.

## Methods

### Literature search

The present systematic review and meta-analysis were conducted according to the previously established protocol. Databases of *PubMed*, *Embase*, *Cochrane Library*, *China National Knowledge Internet* (*CNKI*), *China Biology Medicine disc* (*CBM*), and *Google Scholar* were searched from inception to 1 June 2022 for relevant studies, limited to English and Chinese versions.

The retrieval method was a combination of subject headings and free words. The following index keywords and their similar terms were used in the search: (1) “atrial fibrillation” OR “atrial flutter” OR “atrial flutter” AND (2) “non-vitamin K antagonist oral anticoagulants” OR “direct oral anticoagulants” OR “new oral anticoagulants” OR “rivaroxaban” AND (3) “dialysis” OR “hemodialysis” OR “end-stage kidney disease” OR “stage 5 chronic kidney disease.”

### Study selection

The eligible criteria for studies were as follows: (1) population: NVAF patients with ESKD. ESKD was defined based on a diagnosis of stage 5 chronic kidney disease (CKD) or patients being on regular dialysis; (2) intervention: rivaroxaban, including both low doses of rivaroxaban (10 mg) and recommended dose approved in most districts (20/15 mg); (3) comparison: warfarin or apixaban; (4) outcomes: primary efficacy outcome [composite of stroke and systemic embolism (SSE), ICS, and systemic embolism (SE)], primary safety outcomes [major bleeding, intracranial hemorrhage (ICH), and gastrointestinal bleeding (GIB)]; and (5) study design: cohort studies or RCTs.

The exclusion criteria were as follows: (1) case reports, case series, and systematic reviews; (2) studies that were published only in the form of conference abstracts; (3) published in neither English nor Chinese; and (4) The full text was not available, and the authors could not be contacted.

Two reviewers (SW and YY) independently screened the literature, extracted information, and cross-checked it, and the third party (ZY) negotiated and resolved any doubtful literature. In the first step of literature screening, the *EndNote* literature management software was used to remove duplicates. In the second step, the titles and abstracts were read, and the publications that obviously did not meet the inclusion criteria were eliminated. The third step was to read the full text to determine whether to include it or not.

### Data extraction and quality assessment

Data extraction was performed independently by two authors (JW and YY) using *Excel*, and the extracted information was as follows: the first author, year of publication, types of literature research, source of the research object, interventions, patient age, sex ratio, disease type, CHA2DS2-VASc score, sample size, follow-up time, and outcome indicators.

### Risk of bias assessment

Randomized controlled trials (RCTs) were assessed for risk of bias in the literature using the criteria provided by the Cochrane Collaboration (11). Two researchers independently evaluated, and it includes the following six aspects: (1) generation of random sequences, (2) allocation concealment, (3) blinding of participants and personnel, (4) data integrity, (5) selective reporting, and (6) other biases. According to the risk of bias, outcomes of the assessment include grade A “low risk of bias,” grade B “unclear,” and grade C “high risk of bias.” If all of the risk assessments of the study meet the A-level, the study may have a low risk of corresponding bias and is rated as A-level; if one or more of the risk assessment results of the study are grade B, including the absence of an assessment grade C, then the study has a moderate risk of corresponding bias and is grade B; if one or more of the evaluation items in each risk assessment of the study is grade C, then the study is at high risk of corresponding bias and is grade C.

The cohort study used the Newcastle-Ottawa Scale (NOS) to evaluate the risk of bias, including the following eight aspects (12): (1) representativeness of the exposed cohort, (2) selection of the non-exposed cohort, (3) ascertainment of exposure, (4) whether the expected results were present at the start of the study, (5) comparability of baselines between cohorts, (6) adequacy of outcome evaluation; (7) adequacy of follow-up time, and (8) adequacy of follow-up. The highest score for the fifth item is two points, the rest are one point, and the full score is nine points. The evaluation results were divided into low risk of bias (NOS score ≥7 points), medium risk of bias (4 points ≤ NOS score ≤6 points), and high risk of bias (NOS score ≤3 points).

### Statistical analyses

Meta-analysis was performed using the STATA version 16.0 software. The odds ratio (OR) was selected as the effective index for binary variables. The results were expressed as LogOR value and 95% confidence interval (CI), and the test level was α = 0.05. The heterogeneity analyses among the included studies were determined using the *Q*-test and the heterogeneity index *I*^2^, if *I*^2^ < 50% and *P* > 0.05, there was no statistical heterogeneity, then the fixed-effects model was used for meta-analysis; if *I*^2^ ≥ 50% or *P* < 0.05, there was statistical heterogeneity, then meta-analysis using the random-effects model. Meta-regression was used to analyze the effect of different rivaroxaban doses or control groups on heterogeneity. When meta-analysis included <10 articles, publication bias analysis was not performed. If the number of clinical studies that could be combined was less than 2, only descriptive analysis was performed.

## Results

### Study selection and characteristics

**FIGURE 1:**
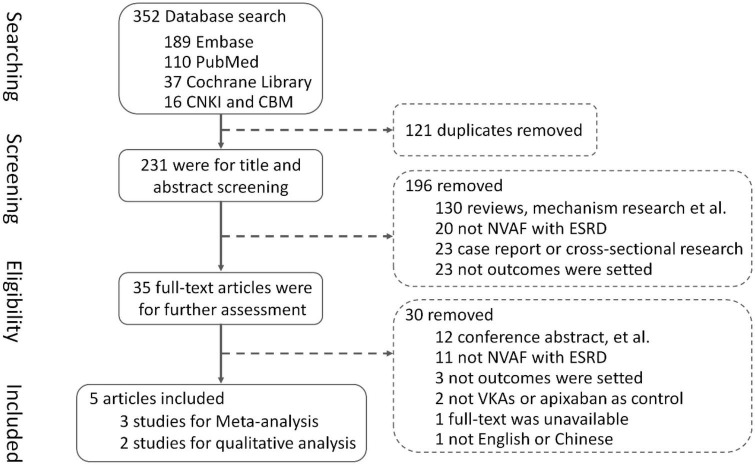
Flow diagram.

In total, the literature search yielded 352 records, and among them, 121 duplicates were removed using EndNote software and screening titles and abstracts. By screening titles and abstracts, 196 articles were excluded which did not meet inclusion criteria or clearly met exclusion criteria, and 35 full-text articles were obtained for further assessment of eligibility. Finally, three studies (13–15) were included for meta-analysis, involving 6,071 patients with NVAF and ESKD, with 1,006 patients using rivaroxaban, 3,229 patients using warfarin, and 1,836 patients using apixaban, and two studies (16, 17) were included for qualitative analysis. A total of 30 articles were excluded for reasons listed in Figure 1.

Among the three studies for meta-analysis, one RCT (15) with off-label low-dose rivaroxaban was conducted in Belgium, two retrospective cohort studies (13, 14) with off-label low-dose rivaroxaban or recommended dose rivaroxaban were implemented in Chinese Taiwan and America. Follow-up times ranged from 10.4 to 36.0 months in these studies.

### Patients characteristics and quality assessment

Detailed patients’ demographics and clinical characteristics of the included studies for meta-analysis are outlined in Table 1. In addition to the RCT, patients with hemodialysis and NVAF were enrolled, and the other two cohort studies included patients with ESKD or dialysis and NVAF. The mean age of patients was approximately 70.0 years, and patients in the RCT (15) seemed elder. The proportion of male was between 25.0 and 60.3%, and the male patient proportion in the RCT study was lower. The mean CHA2DS2-VASc scores were >3 in all articles, and the follow-up duration was about 2.0 years (ranging from 10.4 to 36.0 months).

**TABLE 1:** Demographics and clinical characteristics of the included studies.

References	Study design	Country or region/data source/inclusion period	Drug	Dose(s)	Sample size	Patients	Age, mean ± SD (year)	Male (*n* %)	CHA2DS2-score, mean ± SD	Median follow-up duration (months)	Reported outcomes
Lin et al. (13)	Retrospective cohort study	Taiwan’s Province health insurance research database/2013.2–2017.9	Rivaroxaban	10/15/20 mg/day	173	Patient with ESKD and NVAF	69 ± 11	43	3.8 ± 1.5	19.1	MB, GIB, ICHSSE, ICS, SE
			Warfarin	NA	3185		69 ± 12	49	3.7 ± 1.6	27.4	
Miao et al. (14)	Retrospective	America/US IBM market scan data/2014.1.1–2017.12.31	Rivaroxaban	20 mg/day (71.2%); <20 mg/day (28.8%)	787	ESKD or receiving dialysis with NVAF	70 ± 13	60.3	3.0 ± 1.5	10.4	MB, GIB, ICH SSE, ICS
			Apixaban	10 mg/day (71.2%) <10 mg/day (28.9%)	1836			59.7			
De Vriese et al. (15)	Randomized prospective, open-label	Belgium/randomized/ 2015.2–2019.1	Rivaroxaban	10 mg/day	46	Hemodialysis patients with NVAF	79.9 ± 2.4	35	4.7 ± 1.4	36	MB, GIB, ICH; SSE, ICS, SE
			Warfarin	INR(3-2)	44		80.3 ±3.2	25.0	4.8 ±1.5		

ESKD, end-stage renal disease; NVAF, non-valvular atrial fibrillation; MB, major bleeding; GIB, gastrointestinal bleeding; ICH, hemorrhagic stroke; SSE, composite of stroke and systemic embolism; ICS, ischemic stroke; SE, systemic embolism.

The summary risk of bias in the two included cohort studies [Lin (13) NOS score was 8 and Miao (14) NOS score was 9] was determined as low quality. Risk of bias in the included RCT study was low. The two retrospective cohort studies (13, 14) used inverse probability of treatment weighting (IPTW) based on the propensity score to balance the baseline characteristics of patients receiving warfarin and rivaroxaban to reduce potential selection bias. Detailed risk of bias of meta-analysis is outlined in Table 2.

**TABLE 2:** Risk of bias of the included studies.

References	Selection			Comparability			Outcome		Risk of bias
	**Representativeness of the exposed cohort**	**Selection of the non-exposed cohort**	**Ascertainment of exposure**	**Demonstration that outcome of interest was not present at start of study**	**Comparability of cohorts on the basis of the design or analysis**	**Independent blind assessment**	**Was follow-up long enough for outcomes to occur**	**Adequacy of follow up of cohorts**	
Lin et al. (13)	*	*	*	*	**	–	*	*	Low
Miao et al. (14)	*	*	*	*	**	*	*	*	Low
**References**	**Random sequence generation**	**Allocation concealment**	**Blinding of participants and personnel**	**Blinding of outcome assessment**	**Incomplete outcome data**	**Selective reporting**	**Other biases**		**Risk of bias**
De Vriese et al. (15)	A^a^	A	A	A	A	A	A	A

^a^A: low risk of bias. * Represent the “Selection” and “Outcome” categories. ** Represent the “Comparability.”

### Efficacy and safety of mix-dose rivaroxaban

**FIGURE 2:**
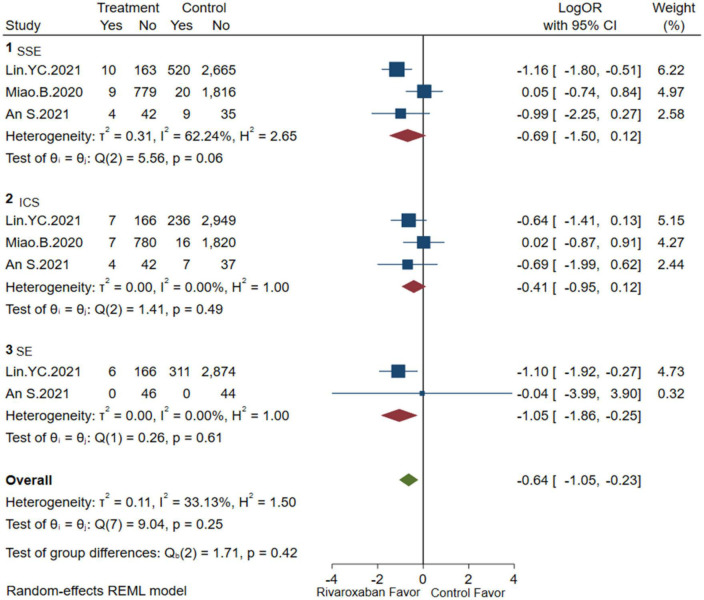
Embolism of mix-dose rivaroxaban (SSE, composite of stroke and systemic embolism; ICS, ischemic stroke; SE, systemic embolism).

**FIGURE 3:**
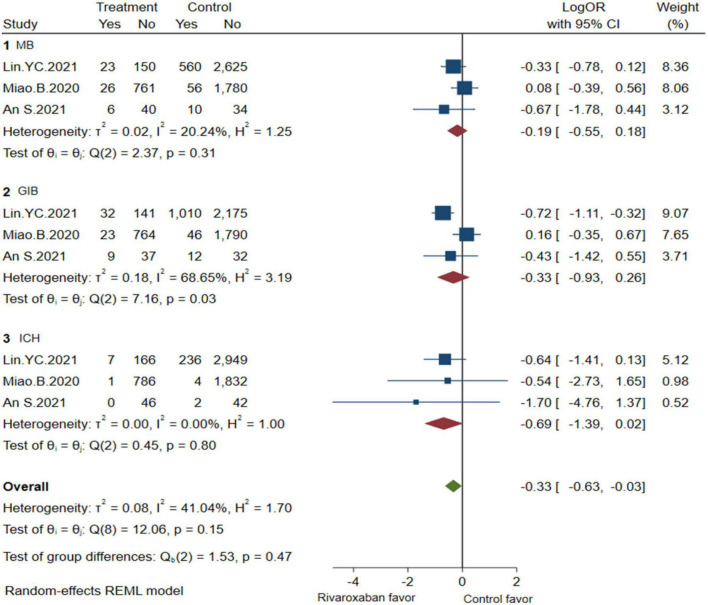
Bleeding of mix-dose rivaroxaban (MB, major bleeding; GIB, gastrointestinal bleeding; ICH, hemorrhagic stroke).

Comprehensive analysis of the efficacy and safety of mixed-dose rivaroxaban and controlled drugs (warfarin or apixaban) in NVAF patients’ companions with ESKD are illustrated in Figures 2, 3. As for the efficacy outcome, a meta-analysis demonstrated that mix-dose rivaroxaban and the control group were found to have no statistical difference in reducing the risk of SSE (3 studies, LogOR: −0.69, 95% CI: −1.50 to 0.12, *P*: 0.06, *I*^2^: 62.2%), ICS (3 studies, LogOR: −0.41, 95% CI: −0.95 to 0.12, *P*: 0.49, *I*^2^: 0.0%), and SE (2 studies, LogOR: −1.05, 95% CI: −1.86 to −0.25, *P*: 0.61, *I*^2^: 0.0%). When considering safety, the treatment of mix-dose rivaroxaban seemed to produce a comparable risk of major bleeding (3 studies, LogOR: −0.19, 95% CI: −0.55 to 0.18, *P*: 0.31, *I*^2^: 20.2%) and ICH (3 studies, LogOR: −0.69, 95% CI: −1.39 to 0.02, *P*: 0.80, *I*^2^: 0.0%), however, conferred a lower risk of GIB (3 studies, LogOR: 0.33, 95% CI: −0.93 to 0.26, *P*: 0.03, *I*^2^: 68.6%) when compared with controlled drugs.

### Efficacy and safety of low-dose rivaroxaban

**FIGURE 4:**
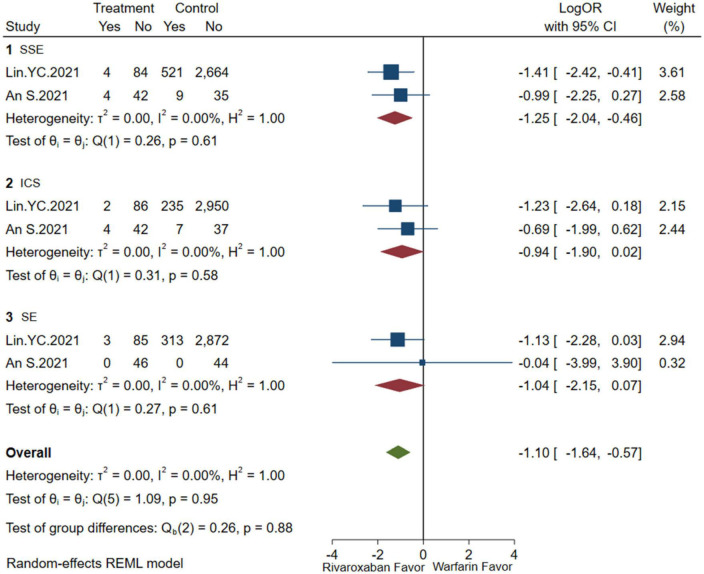
Embolism of low-dose rivaroxaban (SSE, composite of stroke and systemic embolism; ICS, ischemic stroke; SE, systemic embolism).

**FIGURE 5:**
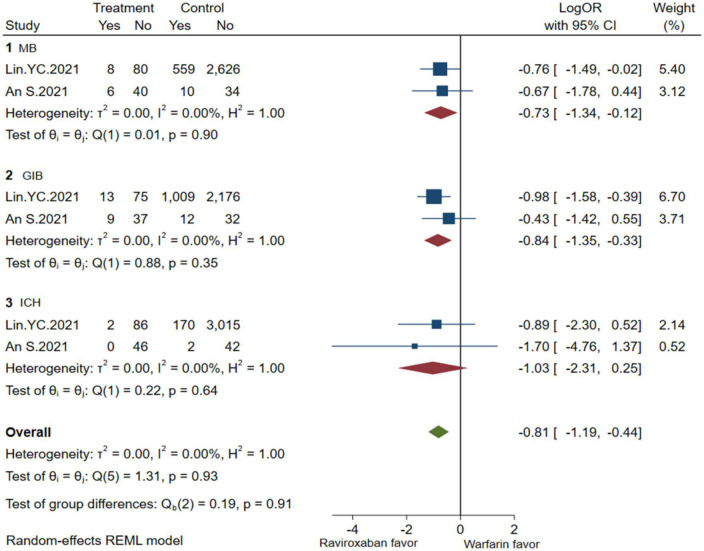
Bleeding of low-dose rivaroxaban (MB, major bleeding; GIB, gastrointestinal bleeding; ICH, hemorrhagic stroke).

A comprehensive analysis of the efficacy and safety of low-dose rivaroxaban and warfarin in patients with NVAF and ESKD is illustrated in Figures 4, 5. In the occurrence of thrombotic events, low-dose rivaroxaban was found to have no statistical difference in reducing the risk of SSE (2 studies, LogOR: −1.25, 95% CI: −2.04 to −0.46, *P*: 0.61, *I*^2^: 0.0%), ICS (2 studies, LogOR: −0.94, 95% CI: −1.90 to 0.02, *P*: 0.58, *I*^2^: 0.0%), and SE (2 studies, LogOR: −1.04, 95% CI: −2.15 to 0.07, *P*: 0.61, *I*^2^: 0.0%). The treatment of low-dose rivaroxaban showed the same risk of major bleeding (2 studies, LogOR: −0.73, 95% CI: −1.34 to −0.12, *P*: 0.90, *I*^2^: 0.0%), GIB (2 studies, LogOR: −0.84, 95% CI: −1.35 to −0.33, *P*: 0.35, *I*^2^:0.0%), and ICH (2 studies, LogOR: −1.03, 95% CI: −2.31 to −0.25, *P*: 0.64, *I*^2^: 0.0%) when compared with warfarin.

### Sensitivity analysis, meta-regression

Meta-regression was used to analyze the heterogeneity. Considering that study design, rivaroxaban dose, and controlled drugs might affect the study results, meta-regression was carried out to observe whether heterogeneity was caused by these factors. For SSE outcome, results showed that study design, rivaroxaban dose, and controlled drug could explain 3.22, 30.4, and 0.0% of heterogeneity, respectively. While for the GIB outcome, results showed that study design, dose, and control drug could explain 35.8, 47.1, and 0.0% of heterogeneity, respectively. It seems that the rivaroxaban dose may be important to the indicators of both SSE and GIB; however, controlled drugs may contribute less to the results.

### The outcome of qualitative literature

Lai et al. (16) conducted a real-world study using data from the Taiwan Provincial Health Insurance System database. A total of 896 patients with NVAF who were receiving dialysis treatment were included in this study, and they were divided into two groups, A and B. Patients in group A were treated with NOACs for anticoagulation therapy, and patients in the other group were treated with warfarin for anticoagulation therapy. It should be noted that nearly 50% of patients in group A received rivaroxaban, and most patients were on low-dose therapy (15/10 mg/day). Two types of study outcomes are as follows: the efficacy index was a composite endpoint of ICS or SE, and the safety index was GIB, ICH, and major hemorrhage. The time of starting anticoagulant therapy (1 June 2012) was the start time of follow-up, and the first occurrence of outcome indicators or reaching the end of the study (31 December 2017) was the end time of follow-up, whichever occurs first. The results of the research showed that group A had no significant difference in the main efficacy indicators and safety indicators (all *P*-values > 0.1) compared with group B (receiving warfarin treatment). However, due to the inability to obtain the specific INR value of patients in the database, the time in therapeutic range (TTR) of patients in group B was not clear, so the actual anticoagulation intensity of warfarin in group B could not be judged. That is to say, the fact that there was no statistical difference in the anticoagulation results between the two groups cannot be excluded due to an insufficient actual anticoagulation intensity of warfarin. In addition, a direct comparison between NOACs is currently lacking, and the remaining patients in group A were anticoagulated with apixaban, dabigatran, or edoxaban. Therefore, the possible differences in efficacy among NOACs may prevent the pooled results from truly reflecting the actual clinical efficacy of rivaroxaban.

Coleman et al. (17) performed a retrospective cohort study by analyzing the US Truven Market Scan database. A total of 6,744 NVAF patients with CKD stages 4–5 or on dialysis (88% with CKD stage 5 or on dialysis) were included in the research; among them were 1,896 patients in group C who took rivaroxaban for anticoagulation therapy (38.7% of patients took a dose < 20 mg/day); 4,848 patients in group D were treated with warfarin. The results of the study indicated that rivaroxaban did not significantly reduce the composite endpoint of stroke or SE (HR = 0.55, 95% CI| : 0.27–1.10) and ICS (HR = 0.67, 95% CI: 0.30–1.50) in group C compared with group D (receiving warfarin). In terms of bleeding control, rivaroxaban in group C reduced total major bleeding by 32% compared with group D, although there was no significant difference between group C and group D in reducing intracranial or GIB. It still cannot be excluded that undetected confounding factors may have influenced the study results, although the research used IPTW to match the included population at baseline to exclude confounding. Furthermore, in spite of the fact that the majority (88%) of the patients included in the study had NVAF with ESKD, we cannot rule out the possibility that the enrollment of additional patients may have influenced the research results.

## Discussion

### Major findings

Based on the available evidence, the study evaluated the efficacy and safety of rivaroxaban among NVAF patients with ESKD. We found that mix-dose rivaroxaban was well matched in risk of embolism (SSE, ICS, and SE) and bleeding (major bleeding and ICH) with controlled drugs (warfarin or apixaban), but demonstrated a lower risk of GIB, which was mainly driven by comparison with warfarin. As for low-dose rivaroxaban (10 mg/day), no significant difference was observed between low-dose rivaroxaban and warfarin in all the clinical outcomes. However, owing to limited studies and considerable heterogeneity, further prospective studies are required to confirm these findings.

### OACs in NVAF patients with ESKD

Whether NVAF patients with ESKD should receive NOACs for stroke prevention is still controversial. When these patients receive anticoagulation therapy, it may be argued that NOACs lack reliable monitoring indicators to guide clinical adjustment of medication, and doctors still tend to give warfarin therapy (18). However, warfarin in patients with CKD should be aware of the following risks. (1) The risk of aggravated vascular calcification: hyperphosphatemia associated with renal failure can stimulate the osteogenic properties of smooth muscle cells, leading to arterial vascular calcification. Warfarin can antagonize vitamin K, inhibit the inhibitory effect of vitamin K-dependent matrix proteins on vascular calcification, and further aggravate the risk of arterial vascular calcification (19). (2) Risk of warfarin-related nephropathy (WRN): WRN is defined as an unexplained increase in serum creatinine of more than 0.3 mg/dl within 1 week after the onset of excessive anticoagulation of warfarin (INR ≥ 3). The mechanism may be excessive anticoagulation with warfarin, glomerular hemorrhage caused by thrombin exhaustion, and red blood cell cast obstruction, leading to acute kidney injury (AKI). The incidence of WRN in patients with CKD was 33%, which was two times as high as that in patients with non-CKD. The occurrence of WRN will also lead to the further deterioration of CKD and increase the mortality of patients with CKD (20). Previous studies have shown that warfarin increases the risk of ICS in patients with NVAF and ESKD, and warfarin can reduce vitamin-dependent protein matrix-carboxylic acid activation, promote plaque formation, and cause calcium ion deposition, and these effects can cause renal vascular calcification or direct kidney damage (21–23), which makes its application in this part of the population skeptical.

The association between chronic inflammation and vascular calcification has been confirmed by current studies, and the most common source of chronic vascular inflammation is atherosclerosis (AS) (24, 25). Factor Xa is a mediator in the initiation and acceleration of AS. Macrophages and vascular smooth muscle cells are involved in the progression of AS. Rivaroxaban can inhibit AS by inhibiting factor Xa-induced protease-activated receptor 2 (PAR2)/Akt/hypoxia-inducible factor 1α (HIF1α) signaling-mediated M1 polarization of macrophages and high δ-like receptor 4 (Dll4) expression, which makes it have a certain vascular protective effect (26).

Authoritative guidelines around the world currently preferentially recommend NOACs for stroke prevention in patients with NVAF, with the publication (27–31) of key RCTs results on NOACs for NVAF. However, considering that NOACs rely on renal excretion to a certain extent and their key RCTs exclude patients with ESKD Currently, only the 2019 AHA/ACC/HRS AF guidelines (32) in the United States state that warfarin or apixaban may be reasonable in patients with NVAF who are dependent on dialysis, while the 2020 version of the guidelines issued (33) by the European Society of Cardiology and the 2021 guidelines issued (34) by the Chinese Society of Atrial Fibrillation believe that the use of NOACs and warfarin for stroke prevention in patients with NVAF and ESKD is insufficient and no clear recommendation has been formed. Individualized treatment is recommended according to the specific conditions of each patient.

Relevant studies of other NOACs in patients with NVAF and ESKD are as follows: (1) Apixaban: A 2017 pharmacokinetic study of apixaban in dialysis patients showed results as follows. In hemodialysis patients, the administration of Apixaban (2.5 mg bid) is comparable to the *in vivo* exposure of the drug at the standard dose (5 mg bid) in patients with preserved renal function, and apixaban (5 mg bid) leads to excessive anticoagulation in hemodialysis patients (35). This study provides a reference for the dosage of apixaban in dialysis patients. In addition, a retrospective cohort study based on the United States Renal Data System, published in the journal *Circulation* in 2018, included 25,523 patients with end-stage renal disease (ESKD) (2,351 patients in the apixaban group and 23,172 patients in the warfarin group). The findings indicated little difference in the risk of stroke/systemic embolism between apixaban and warfarin (HR, 0.88; 95% CI, 0.69–1.12; *P* = 0.29); however, apixaban is associated with a significantly lower risk of major bleeding (HR, 0.72; 95% CI, 0.59–0.87; *P* ≤ 0.001). Sensitivity analyses showed a significantly lower risk of stroke/systemic embolism and death with the standard dose of apixaban (5 mg bid) compared with the reduced dose of apixaban (2.5 mg bid) (stroke/systemic embolism: HR, 0.61; 95% CI, 0.37–0.98; *P* = 0.04; death: HR, 0.64; 95% CI, 0.45–0.92; *P* = 0.01), risks for stroke/systemic embolism and death were significantly lower compared with warfarin (stroke/systemic embolism: HR, 0.64; 95% CI, 0.42–0.97; *P* = 0.04; death: HR, 0.63; 95% CI, 0.46–0.85; *P* = 0.003) (36). A reduced dose of apixaban (2.5 mg bid) is recommended considering that apixaban may increase the risk of major bleeding. (2) Dabigatran etexilate: A study published in 2014 on the drug metabolism of dabigatran etexilate in dialysis patients showed that after a single administration of dabigatran etexilate before dialysis, the average peak time of free blood concentration was 2 h (1–3 h), and the average peak concentration was 95.5 ± 33.4 ng/ml. The mean elimination half-lives of hemodialysis and after hemodialysis were 2.6 ± 1.3 h and 30.2 ± 7.8 h, respectively, and dabigatran was effectively removed by hemodialysis with a clearance rate of 0.63 ± 0.07 (37). A study based on the North American Health Care Database [Fresenius Medical Care North America (FMCNA)] that collected and analyzed the clinical use of dabigatran etexilate in dialysis patients showed that dabigatran etexilate was associated with a higher risk of hospitalization or bleeding death (RR, 1.48; 95% CI, 1.21–1.81; *P* = 0.0001) compared with warfarin and a greater risk of hemorrhagic death (RR, 1.78; 95% CI, 1.18–2.68; *P* = 0.006). As few stroke and arterial embolic events were studied, no meaningful differences between drug groups were detected (38). (3) Unfortunately, for all we know, no studies related to edoxaban were identified so far.

### Rivaroxaban in NVAF patients with ESKD

The pharmacokinetics of rivaroxaban (15 mg/day) in patients undergoing dialysis and patients with normal renal function (CL_*CR*_ ≥ 80 ml/min) have been indicated in the literature (39). The results are declared as follows. (1) Group E (patients receiving dialysis) were administered 2 h before hemodialysis, with an average C_max_ of 194 ng/ml, and administered 3 h after hemodialysis, with an average C_max_ of 247 ng/ml, and group F (normal renal function) with an average C_max_ of 210 ng/ml, indicating that renal function has less effect on rivaroxaban C_max_. (2) Compared with group F, when administered 3 h after dialysis, the mean AUC in group E was 2,907 ng⋅h/ml, representing a 56% increase in systemic exposure, similar to the 52% increase in AUC in patients with moderate renal insufficiency or the 64% increase in AUC in patients with severe renal insufficiency (40). (3) Dosing 4 h before dialysis resulted in only a 5% reduction in plasma AUC compared to dosing 3 h after dialysis, suggesting that dialysis had less effect on rivaroxaban exposure *in vivo*. This study demonstrated comparable drug exposure with a single dose of rivaroxaban 15 mg in patients with moderate, severe, or ESKD.

Only one RCT (15) reported the use of rivaroxaban for stroke prevention in patients with NVAF and ESKD; an additional 18-month follow-up period was extended after the completion of the initial 18-month follow-up study. The study showed that rivaroxaban (10 mg/day) could improve the clinical efficacy compared with warfarin (INR 2.0–3.0), which means rivaroxaban could reduce the risk of fatal cardiovascular disease and a composite of non-fatal stroke, cardiac events, and other vascular events (41). Moreover, in two other cohort studies based on the Medicare database, Miao et al.’s (14) cohort study reported no difference between apixaban and rivaroxaban, Lin. YC’s (13) cohort study reported SSE was significantly lower in the rivaroxaban group and low-dose rivaroxaban group (primarily driven by ICS) than in the warfarin group; the major bleeding risk was similar between rivaroxaban and warfarin users, and further analysis showed GIB risk was significantly lower in the rivaroxaban group and low-dose rivaroxaban group than in the warfarin group.

As in this meta-analysis, rivaroxaban carried out comparable embolism events and bleeding; forest maps show that rivaroxaban may be superior to warfarin, especially in reducing GIB. According to previous studies, rivaroxaban was associated with more GIB events, and the results of this study may have the following reasons. (1) This study was a retrospective cohort study. Although the baseline was matched by the propensity score matching method, the influence of other unconsidered confounding factors on the results cannot be excluded, and there may be the possibility of information collection bias. (2) There were 173 cases in the rivaroxaban group and 3,185 cases in the warfarin group, and the proportion of patients in the two groups was significantly different, which may lead to insufficient efficacy of statistical results. (3) The use of proton pump inhibitors could not be obtained from the literature during the treatment of patients with oral anticoagulant drugs. Considering the actual clinical medication, patients taking rivaroxaban tend to use proton pump inhibitors, and it cannot be excluded that the proportion of patients taking rivaroxaban combined with proton pump inhibitors is higher, which leads to a reduction in GIB events in this group. (4) Patients with ESKD are prone to excessive anticoagulation (abnormally elevated INR) due to fluctuating INR during warfarin therapy. The TTR target value is ≥70% (42) in patients with CKD. The higher rate of GIB in the warfarin group due to substandard TTR cannot be ruled out.

Considering 10 mg of rivaroxaban administered to chronic hemodialysis patients had similar pharmacokinetic outcomes to 20 mg given to healthy volunteers (43). We suggest that rivaroxaban 10 mg may be an alternative in such kind of population. However, the increased risk of bleeding, in particular of hemorrhagic stroke should take into account when assessing the overall effect of VKA in hemodialysis patients. Results from a large phase III clinical trial of rivaroxaban showed a significant reduction in ICH (0.5 vs. 0.7%, *P* = 0.02) and fatal bleeding (0.2 vs. 0.5%, *P* = 0.003) in rivaroxaban compared with warfarin for stroke prophylaxis with AF (31). In 2016, Joji Hagii et al. (44) initiated rivaroxaban treatment in 65 patients with acute cardiogenic stroke 5 days after onset. By measuring anti-Xa activity, assessing plasma prothrombin fragment 1 + 2 (F1 + 2), prothrombin time (PT), and rivaroxaban concentrations, among the findings, Rivaroxaban maintained normal thrombin production even at peak concentrations, while therapeutic levels of warfarin inhibited thrombin production, and the study may partly explain the different outcomes in patients with bleeding events. However, patients with ESKD were excluded from the above clinical trials, and the results cannot be directly extrapolated to this population.

In addition, considering that the dose-response relationship of anticoagulants is related to race, this is related to the anticoagulant efficacy of the drug itself and the pharmacokinetics *in vivo*. The dose stratification in this study was based on the FDA-approved dose of rivaroxaban for NVAF (20 mg/day when CrCl ≥ 50 ml/min, 15 mg/day when 15 ml/min ≤ CrCl < 50 ml/min). With the publication of J-ROCKET study data (30), rivaroxaban was prescribed at 15 mg/day for CrCl ≥ 50 ml/min and at 10 mg/day for 30 ml/min ≤ CrCl < 50 ml/min for patients with NVAF, which is basically consistent with the results of the global ROCKET AF trial (31), but the incidence of GIB in the rivaroxaban group in the J-ROCKET study was lower than in the warfarin group, and unlike the global ROCKET AF trial, this difference does not rule out racial differences. Based on this result, this dose has been approved for stroke prophylaxis with NVAF in Japan and Taiwan, China. However, considering that rivaroxaban 10 mg/day has limited data on the Chinese mainland, this is still consistent with FDA approval. One (13) of the three studies analyzed quantitatively was from an Asian population in Taiwan, China, and the other two (14, 15) were from a European population in the United States and Belgium. Although there was no significant heterogeneity in embolic and bleeding events at mixed and low doses in this study and only high heterogeneity (62.2 and 68.6%) between SSE and GIB at mixed doses, it was not ruled out that differences between different ethnic groups were not manifested due to the small number of studies. Similarly, for patients with NVAF and ESKD, it is also necessary to consider the different dose requirements caused by different races when taking rivaroxaban.

The study population included in this study was NVAF patients with ESKD, but there was only one RCT with a small sample size, and the rest were cohort studies, which also limited the extrapolation of the results. Meta-analysis achieves the purpose of increasing the sample size and improving the test power by combining similar studies. However, due to the limitations of current clinical research, the number of articles included in this meta-analysis is small, especially given the small number of patients in the rivaroxaban group, so RCTs with large sample sizes are still needed to confirm the study results.

Very low-dose rivaroxaban (2.5 mg two times daily) has been recommended for patients with acute coronary syndromes to improve survival, primarily based on the results of the ATLAS ACS 2–TIMI51 study, which was published in the *New England Journal* in 2012 (45). The study also prescribed a 10 mg/day dosing regimen (5 mg/time two times daily), but the guidelines did not recommend a 10 mg/day regimen for rivaroxaban because it did not significantly reduce the risk of death from cardiovascular causes compared with placebo and increased bleeding compared with rivaroxaban 5 mg/day dosing regimen. However, the study was conducted with rivaroxaban in combination with antiplatelet drugs (98.7% in combination with aspirin), and the study excluded patients with CrCl < 30 ml/min, and the cardiovascular benefit and bleeding risk of rivaroxaban 5 mg/day alone in patients with NVAF and ESKD are uncertain.

### Study strengths and limitations

To the best of our knowledge, this is the first study that focuses on the benefits and harms of NVAF patients with ESKD treated with rivaroxaban. According to the retrieval and screening of published literature systematically and combined analysis of relevant studies, we provide a reference for clinicians when prescribing anticoagulants to such kind of a population. However, some limitations need to be acknowledged. First, there were only three studies that could be quantitatively analyzed, and only one was an RCT. Although the two retrospective cohort studies balanced the baseline characteristics of patients by using *IPTW*, the risk of bias in the included studies was all determined to be low. Second, labile INR data were not accessible in the cohort study, which used warfarin as the control drug. Ultimately, considering the small number of patients included in this study, the results of this study still need to be further validated in NVAF patients with ESKD.

## Data availability statement

The original contributions presented in this study are included in the article/supplementary material, further inquiries can be directed to the corresponding authors.

## Author contributions

ZY, SW, and ZZ conceived the work and directed experiments. JW, YY, and SW searched databases and screened all titles and abstracts. FR and TC conducted study selection, data extraction, and quality assessment. ZY, JW, and SW drafted the first two editions of this manuscript. All authors agreed to this version of the manuscript.

## References

[1] Benjamin EJ Muntner P Alonso A Bittencourt MS Callaway CW Carson AP Heart disease and stroke statistics-2019 update: a report from the American Heart Association. *Circulation.* (2019) 139:e56–528. 30700139 10.1161/CIR.0000000000000659

[2] Alonso A Lopez FL Matsushita K Loehr LR Agarwal SK Chen LY Chronic kidney disease is associated with the incidence of atrial fibrillation: the Atherosclerosis Risk in Communities (ARIC) study. *Circulation.* (2011) 123:2946–53. 10.1161/CIRCULATIONAHA.111.020982 21646496 PMC3139978

[3] Soliman EZ Prineas RJ Go AS Xie D Lash JP Rahman M Chronic kidney disease and prevalent atrial fibrillation: the Chronic Renal Insufficiency Cohort (CRIC). *Am Heart J.* (2010) 159:1102–7. 20569726 10.1016/j.ahj.2010.03.027 PMC2891979

[4] Genovesi S Pogliani D Faini A Valsecchi MG Riva A Stefani F Prevalence of atrial fibrillation and associated factors in a population of long-term hemodialysis patients. *Am J Kidney Dis.* (2005) 46:897–902. 10.1053/j.ajkd.2005.07.044 16253730

[5] Wizemann V Tong L Satayathum S Disney A Akiba T Fissell RB Atrial fibrillation in hemodialysis patients: clinical features and associations with anticoagulant therapy. *Kidney Int.* (2010) 77:1098–106. 10.1038/ki.2009.477 20054291

[6] Ramagopalan SV Stamp E Sammon CJ Besford M Carroll R Mehmud F Renal function and oral anticoagulant treatment of incident non-valvular atrial fibrillation: a retrospective study. *Future Cardiol.* (2019) 15:301–9. 10.2217/fca-2019-0012 31140872

[7] van Zyl M Abdullah HM Noseworthy PA Siontis KC. Stroke prophylaxis in patients with atrial fibrillation and end-stage renal disease. *J Clin Med.* (2020) 9:123. 10.3390/jcm9010123 31906546 PMC7019832

[8] Randhawa MS Vishwanath R Rai MP Wang L Randhawa AK Abela G Association between use of warfarin for atrial fibrillation and outcomes among patients with end-stage renal disease: a systematic review and meta-analysis. *JAMA Netw Open.* (2020) 3:e202175. 10.1001/jamanetworkopen.2020.2175 32250434 PMC7136833

[9] Yang F Chou D Schweitzer P Hanon S. Warfarin in haemodialysis patients with atrial fibrillation: what benefit? *Europace.* (2010) 12:1666–72. 10.1093/europace/euq387 21045011

[10] Chan KE Lazarus JM Thadhani R Hakim RM. Warfarin use associates with increased risk for stroke in hemodialysis patients with atrial fibrillation. *J Am Soc Nephrol.* (2009) 20:2223–33. 10.1681/ASN.2009030319 19713308 PMC2754104

[11] Higgins JP Altman DG Gotzsche PC Juni P Moher D Oxman AD The cochrane collaboration’s tool for assessing risk of bias in randomised trials. *BMJ.* (2011) 343:d5928. 10.1136/bmj.d5928 22008217 PMC3196245

[12] Islam MA Khandker SS Alam F Kamal MA Gan SH. Genetic risk factors in thrombotic primary antiphospholipid syndrome: a systematic review with bioinformatic analyses. *Autoimmun Rev.* (2018) 17:226–43. 10.1016/j.autrev.2017.10.014 29355608

[13] Lin YC Chen BL Shih CM Lin FY Chen CW Hsu CY Effectiveness and safety of rivaroxaban versus warfarin in Taiwanese patients with end-stage renal disease and nonvalvular atrial fibrillation: a real-world nationwide cohort study. *PLoS One.* (2021) 16:e0249940. 10.1371/journal.pone.0249940 33831130 PMC8031437

[14] Miao B Sood N Bunz TJ Coleman CI. Rivaroxaban versus apixaban in non-valvular atrial fibrillation patients with end-stage renal disease or receiving dialysis. *Eur J Haematol.* (2020) 104:328–35. 10.1111/ejh.13383 31925840

[15] De Vriese AS Caluwe R Van Der Meersch H De Boeck K De Bacquer D. Safety and efficacy of vitamin k antagonists versus rivaroxaban in hemodialysis patients with atrial fibrillation: a multicenter randomized controlled trial. *J Am Soc Nephrol.* (2021) 32:1474–83. 10.1681/ASN.2020111566 33753537 PMC8259651

[16] See LC Lee HF Chao TF Li PR Liu JR Wu LS Effectiveness and safety of direct oral anticoagulants in an asian population with atrial fibrillation undergoing dialysis: a population-based cohort study and meta-analysis. *Cardiovasc Drugs Ther.* (2021) 35:975–86. 10.1007/s10557-020-07108-4 33211254

[17] Coleman CI Kreutz R Sood NA Bunz TJ Eriksson D Meinecke AK Rivaroxaban versus warfarin in patients with nonvalvular atrial fibrillation and severe kidney disease or undergoing hemodialysis. *Am J Med.* (2019) 132:1078–83. 10.1016/j.amjmed.2019.04.013 31054829

[18] Chao TF Chen SA. Risk of ischemic stroke and stroke prevention in patients with atrial fibrillation and renal dysfunction. *J Atr Fibrillation.* (2015) 8:1196. 10.4022/jafib.1196 PMC5135110 27957171

[19] El-Abbadi M Giachelli CM. Mechanisms of vascular calcification. *Adv Chronic Kidney Dis.* (2007) 14:54–66. 10.1053/j.ackd.2006.10.007 17200044

[20] Brodsky SV Satoskar A Chen J Nadasdy G Eagen JW Hamirani M Acute kidney injury during warfarin therapy associated with obstructive tubular red blood cell casts: a report of 9 cases. *Am J Kidney Dis.* (2009) 54:1121–6. 10.1053/j.ajkd.2009.04.024 19577348

[21] Holden RM Sanfilippo AS Hopman WM Zimmerman D Garland JS Morton AR. Warfarin and aortic valve calcification in hemodialysis patients. *J Nephrol.* (2007) 20:417–22. 17879207

[22] Wang Y Zhang W Zhang Y Yang Y Sun L Hu S VKORC1 haplotypes are associated with arterial vascular diseases (stroke, coronary heart disease, and aortic dissection). *Circulation.* (2006) 113:1615–21. 10.1161/CIRCULATIONAHA.105.580167 16549638

[23] Chatrou ML Winckers K Hackeng TM Reutelingsperger CP Schurgers LJ. Vascular calcification: the price to pay for anticoagulation therapy with vitamin K-antagonists. *Blood Rev.* (2012) 26:155–66. 10.1016/j.blre.2012.03.002 22520397

[24] Aikawa E Nahrendorf M Figueiredo JL Swirski FK Shtatland T Kohler RH Osteogenesis associates with inflammation in early-stage atherosclerosis evaluated by molecular imaging in vivo. *Circulation.* (2007) 116:2841–50. 10.1161/CIRCULATIONAHA.107.732867 18040026

[25] Abdelbaky A Corsini E Figueroa AL Fontanez S Subramanian S Ferencik M Focal arterial inflammation precedes subsequent calcification in the same location: a longitudinal FDG-PET/CT study. *Circ Cardiovasc Imaging.* (2013) 6:747–54. 10.1161/CIRCIMAGING.113.000382 23833282

[26] Ma Y Zhang Y Qiu C He C He T Shi S Rivaroxaban suppresses atherosclerosis by inhibiting FXa-induced macrophage M1 polarization-mediated phenotypic conversion of vascular smooth muscle cells. *Front Cardiovasc Med.* (2021) 8:739212. 10.3389/fcvm.2021.739212 34869643 PMC8634446

[27] Connolly SJ Ezekowitz MD Yusuf S Eikelboom J Oldgren J Parekh A Dabigatran versus warfarin in patients with atrial fibrillation. *N Engl J Med.* (2009) 361:1139–51. 10.1056/NEJMoa0905561 19717844

[28] Giugliano RP Ruff CT Braunwald E Murphy SA Wiviott SD Halperin JL Edoxaban versus warfarin in patients with atrial fibrillation. *N Engl J Med.* (2013) 369:2093–104. 10.1056/NEJMoa1310907 24251359

[29] Granger CB Alexander JH McMurray JJ Lopes RD Hylek EM Hanna M Apixaban versus warfarin in patients with atrial fibrillation. *N Engl J Med.* (2011) 365:981–92. 10.1056/NEJMoa1107039 21870978

[30] Hori M Matsumoto M Tanahashi N Momomura S Uchiyama S Goto S Rivaroxaban vs. warfarin in Japanese patients with atrial fibrillation - the J-ROCKET AF study. *Circ J.* (2012) 76:2104–11. 10.1253/circj.cj-12-0454 22664783

[31] Patel MR Mahaffey KW Garg J Pan G Singer DE Hacke W Rivaroxaban versus warfarin in nonvalvular atrial fibrillation. *N Engl J Med.* (2011) 365:883–91. 10.1056/NEJMoa1009638 21830957

[32] January CT Wann LS Calkins H Chen LY Cigarroa JE Cleveland JC Jr 2019 AHA/ACC/HRS focused update of the 2014 AHA/ACC/HRS guideline for the management of patients with atrial fibrillation: a report of the American College of Cardiology/American Heart Association task force on clinical practice guidelines and the Heart Rhythm Society in collaboration with the Society of Thoracic Surgeons. *Circulation.* (2019) 140:e125–51. 10.1161/CIR.0000000000000665 30686041

[33] Hindricks G Potpara T Dagres N Arbelo E Bax JJ Blomstrom-Lundqvist C 2020 ESC guidelines for the diagnosis and management of atrial fibrillation developed in collaboration with the European Association for Cardio-Thoracic Surgery (EACTS): the task force for the diagnosis and management of atrial fibrillation of the European Society of Cardiology (ESC) developed with the special contribution of the European Heart Rhythm Association (EHRA) of the ESC. *Eur Heart J.* (2021) 42:373–498. 10.1093/eurheartj/ehab648 32860505

[34] LoPresti P. HDAC6 in diseases of cognition and of neurons. *Cells.* (2020) 10:12. 10.3390/cells10010012 33374719 PMC7822434

[35] Mavrakanas TA Samer CF Nessim SJ Frisch G Lipman ML. Apixaban pharmacokinetics at steady state in hemodialysis patients. *J Am Soc Nephrol.* (2017) 28:2241–8. 10.1681/ASN.2016090980 28302754 PMC5491286

[36] Siontis KC Zhang X Eckard A Bhave N Schaubel DE He K Outcomes associated with apixaban use in patients with end-stage kidney disease and atrial fibrillation in the United States. *Circulation.* (2018) 138:1519–29. 10.1161/CIRCULATIONAHA.118.035418 29954737 PMC6202193

[37] Wilson JA Goralski KB Soroka SD Morrison M Mossop P Sleno L An evaluation of oral dabigatran etexilate pharmacokinetics and pharmacodynamics in hemodialysis. *J Clin Pharmacol.* (2014) 54:901–9. 10.1002/jcph.335 24846496

[38] Chan KE Edelman ER Wenger JB Thadhani RI Maddux FW. Dabigatran and rivaroxaban use in atrial fibrillation patients on hemodialysis. *Circulation.* (2015) 131:972–9. 10.1161/CIRCULATIONAHA.114.014113 25595139 PMC4363265

[39] Dias C Moore KT Murphy J Ariyawansa J Smith W Mills RM Pharmacokinetics, pharmacodynamics, and safety of single-dose rivaroxaban in chronic hemodialysis. *Am J Nephrol.* (2016) 43:229–36. 10.1159/000445328 27100875

[40] Kubitza D Becka M Mueck W Halabi A Maatouk H Klause N Effects of renal impairment on the pharmacokinetics, pharmacodynamics and safety of rivaroxaban, an oral, direct Factor Xa inhibitor. *Br J Clin Pharmacol.* (2010) 70:703–12. 10.1111/j.1365-2125.2010.03753.x 21039764 PMC2997310

[41] De Vriese AS Caluwe R Pyfferoen L De Bacquer D De Boeck K Delanote J Multicenter randomized controlled trial of vitamin K antagonist replacement by rivaroxaban with or without vitamin K2 in hemodialysis patients with atrial fibrillation: the valkyrie study. *J Am Soc Nephrol.* (2020) 31:186–96. 10.1681/ASN.2019060579 31704740 PMC6935010

[42] Yuexin C Liping D Lixin Z Yuehong Z Peripheral Vascular Disease Professional Committee of Chinese Society of Microcirculation. (2021). Expert consensus on microcirculation in anticoagulation therapy for venous thromboembolism combined with chronic kidney disease. *J Vasc Endovascular Surg.* 10.19418/j.cnki.issn2096-0646.2021.01.01.

[43] De Vriese AS Caluwe R Bailleul E De Bacquer D Borrey D Van Vlem B Dose-finding study of rivaroxaban in hemodialysis patients. *Am J Kidney Dis.* (2015) 66:91–8. 25804678 10.1053/j.ajkd.2015.01.022

[44] Hagii J Tomita H Metoki N Tamai Y Saito S Shiroto H Effect of rivaroxaban on prothrombin fragment 1+2 compared with warfarin in patients with acute cardioembolic stroke: insight from its serial measurement. *Thromb Res.* (2016) 148:9–14. 10.1016/j.thromres.2016.10.011 27764730

[45] Mega JL Braunwald E Wiviott SD Bassand JP Bhatt DL Bode C Rivaroxaban in patients with a recent acute coronary syndrome. *N Engl J Med.* (2012) 366:9–19. 10.1056/NEJMoa1112277 22077192

